# An investigation of university students' perspectives on combating addiction: a qualitative study

**DOI:** 10.3389/fpubh.2026.1860766

**Published:** 2026-06-18

**Authors:** Şafak Yalçın Şahiner, Dilek Baysal, Abdullah Yildiz, Beril Boran

**Affiliations:** 1 Department of Psychiatry, Faculty of Medicine, Ankara University, Ankara, Türkiye; 2 Combating for Addiction Application and Research Center, Ankara University, Ankara, Türkiye; 3 Department of History of Medicine and Ethics, Faculty of Medicine, Ankara University, Ankara, Türkiye; 4 Institute of Forensic Sciences, Ankara University, Ankara, Türkiye

**Keywords:** combating addiction, perspectives, policy, prevention, qualitative study, university students, youth

## Abstract

**Introduction:**

Assessing the knowledge, awareness, perspectives, and desires for active involvement among youth regarding the combating of addiction and related policies holds a significant position within addiction prevention and intervention efforts. This study aims to evaluate university students' awareness, thoughts, and recommendations concerning the combating of addiction and its associated policies.

**Methods:**

A qualitative research design was adopted for this study. The study group consisted of 119 undergraduate students enrolled at a university in Türkiye. Data were collected via an online survey form developed by the researchers, which enabled participants to provide their own written expressions. During the data analysis process, a descriptive thematic content analysis approach was employed, in alignment with the descriptive qualitative research design.

**Results:**

Our results demonstrated that university students possess a multidimensional perspective on the combating of addiction. It was observed that students support not only macro-level structural interventions but also individual-centered strategies. The most frequently emphasized themes were “Prohibitions, Sanctions, and Monitoring Mechanisms” and “Education and Awareness-Oriented Approaches.” A significant finding of our study is that students' knowledge regarding existing policies is considerably insufficient.

**Conclusion:**

Our study indicates that involving youth and university students in the process of combating and preventing addiction is crucial for ensuring that strategies achieve greater effectiveness.

## 1 Introduction

Addiction, which has significant effects on the young population, is a public health issue that threatens both individual and societal health and the social fabric. In particular, it is observed that risk-taking behavior increases during adolescence and young adulthood, and consequently, both substance and non-substance-related addictions tend to begin more frequently during this period. For instance, the United Nations Office on Drugs and Crime reports a surge in substance use throughout young adulthood (1). Similarly, the World Health Organization observes that complications arising from substance dependence are disproportionately high among individuals aged 15–29 (2). Furthermore, global data demonstrate that this age group exhibits higher rates of alcohol, tobacco, and illicit drug consumption relative to the general population (3). From this standpoint, this demographic represents the most vulnerable and at-risk segment of society regarding the development of addiction.

The university years (ages 18–24), a key stage of young adulthood, constitute a critical period that warrants careful consideration with regard to substance use. University students may engage in a range of risky behaviors, including substance misuse, substance use disorders, and behavioral addictions. Contributing factors may include reduced parental supervision during university, an increased sense of autonomy, heightened peer influence, the presence of academic stress, and changes in the social environment (4).

The development and implementation of effective policies to address addiction are essential for protecting young people and supporting their recovery. Prevention programmes implemented in school and university settings have been shown to be effective in reducing addiction among young people (5, 6). Moreover, it is important not only to consider the content of such policies but also to incorporate the perspectives and expectations of their target population. In particular, as young people often have closer exposure to environments and factors associated with addiction and other risky behaviors, and are able to observe these contexts directly, they can provide valuable insights for the prevention of addiction.

A study conducted by Terzioglu and Ayhan reported that 63.9% of students perceived themselves as being addicted to technology, particularly smartphones and social media (7). This finding underscores the need to address all forms of addiction within prevention efforts and to do so from the perspective of young people. Furthermore, evidence suggests that understanding young people's awareness of addiction and their risk perceptions, as well as incorporating the views, ideas, and solutions they propose, enhances the effectiveness of the policy development process (8).

Young people's awareness of the risks associated with substance use and behavioral addictions can shape their attitudes toward addiction. In particular, greater awareness and knowledge of health risks can lead to the recognition of addiction as a serious problem within this age group and can increase their willingness to engage in preventive measures. For example, perceiving internet addiction as a health risk makes young people more likely to exercise self-regulation in their internet use (9). This suggests that increasing knowledge about the risks and harms of addiction, along with initiatives designed for this purpose, can positively influence attitudes and behaviors among young people. Similarly, studies have shown that interventions targeting social media addiction can significantly improve mental health and self-esteem among young adults (10).

Peer relationships and social support are another key factor shaping attitudes and perspectives toward addiction. Young adults often seek social approval and a sense of belonging, which may lead them to experiment with substances or engage in addictive behaviors. Prevention programmes that emphasize peer support and community engagement can empower young people and support their efforts to address addiction. A study has shown that interventions promoting participation in extracurricular activities and community service reduce rates of substance use and other risky behaviors among young people (11). Furthermore, research indicates that establishing and maintaining supportive relationships with young people can reduce the risk of substance misuse (12).

Through this study, the perception of addiction among young people is addressed beyond individual expression, within a multi-layered dimension. In this context, the Socio-Ecological Model, which is widely utilized in addiction studies, has been taken as a foundation. Developed by Urie Bronfenbrenner, this model describes the reflections of diverse social influences and interconnected environmental interactions on individuals (13). With this model, it is possible to understand how individual attitudes (micro) as well as family and social circles (mezo), the societal framework (exo), and state policies (macro) influence one another, and consequently, how individuals develop in conjunction with these structures (14). Within the family and social environment layer of this model, the immediate social circle-particularly peers-is recognized as a mechanism that significantly influences an individual's risk perception. This perspective is not limited solely to this model; it is also supported by Bandura's Social Learning Theory, which is frequently encountered in addiction studies (15). From this perspective, the importance of querying students on both the individual dimensions of combating addiction and the macro-level systems becomes evident. Consequently, as in our study, it allows for the formulation of a hypothesis that addressing addiction requires not only individual-level interventions but also a multi-systemic coordination.

Assessing university students' knowledge, awareness, perspectives, and willingness to engage actively in efforts to address addiction and related policies plays a crucial role in addiction prevention and control. The aim of this study is to evaluate university students' awareness, perspectives, and suggestions regarding the fight against addiction and related policies. It seeks to inform the development of more effective policy recommendations and prevention strategies by analyzing the current situation, needs, and perspectives on the issue.

## 2 Methods

This study is a qualitative research project designed to explore university students' views, experiences, and recommendations regarding policies to address addiction. Although the study is qualitative in nature, a descriptive qualitative approach was adopted, as the data were collected through a survey. Descriptive qualitative research aims to present participants' experiences, perceptions, and perspectives in their own words. This approach is particularly suitable for studies that require the systematic collection of data from a broad sample on a specific topic and the presentation of these data within a descriptive framework (16). This study is reported following the Standards for Reporting Qualitative Research (SRQR), which provides a design-appropriate reporting framework for open-ended written-survey designs (17).

The research and data collection process was conducted following approval from the Ankara University Ethics Committee (Date: 24 April 2025, No.: 2025/329). Participants who provided informed consent were granted access to the survey content.

### 2.1 Participants

The study group consisted of 119 undergraduate students enrolled at a university in Türkiye. The sample size of our study was determined within the framework of the “Information Power” model proposed by Malterud et al. (18). The objective of our research is to provide a broad landscape mapping regarding the fight against addiction and related policies. Therefore, the sample was kept extensive to enhance information power. Furthermore, by enabling the participation of students from different ages and faculties, the study aimed to achieve a diversity of perspectives.

Participants were selected using a convenience sampling technique, one of the purposive sampling methods. During this process, the necessary permissions were obtained from the university; the online version of the data collection form was distributed to the relevant faculties via the Student Affairs Office and subsequently to students, and the form was administered. Participation in the study was voluntary; participants were granted access to the data collection instrument after providing informed consent online. “The study group consisted of students enrolled at a university in Türkiye during the Spring Semester of the 2024–2025 academic year. The limited number of studies evaluating university students' views, awareness, and proposed solutions regarding the fight against addiction, together with the significant role university students play in this effort, informed the selection of this sample. The convenience sampling method was employed for the study sample. Convenience sampling offers practical advantages among qualitative research methods (19); however, it has been noted that data obtained from samples created using this method may lack diversity (20). Nevertheless, it was planned to partially address this limitation by reaching a relatively broader sample through online data collection.

### 2.2 Data collection tools and the data collection process

Data were collected through an online questionnaire developed by the researchers, which enabled participants to provide written responses in their own words. The use of online data collection methods has recently expanded to qualitative research as well (21). In our study, rather than obtaining the experiences of participants in depth and detail, an online open-ended survey method was preferred. While this method involves recognized limitations regarding dynamic probing and the depth of individual narratives, in our study this trade-off was a deliberate methodological choice rather than a concession. This is because the aim of our research was to generate data specifically for policy studies rather than obtaining deep data from a narrow group of students. Accordingly, priority was given to conducting a broad landscape mapping. Consequently, this method does not analyze the existential processes of the participants; instead, it aims to document the holistic trends, dilemmas, and solution proposals of students regarding policies and best practices. On the other hand, they allow students and young people, who constitute our participant group, to express their views on addiction and policy issues anonymously, without feeling under pressure, freely, and in their own words. Furthermore, these surveys provide the opportunity to reach a larger audience. Thus, studies that gather the views of such broad masses can add different dimensions and provide a highly effective direction to policy studies.

The questionnaire comprised five open-ended questions following the demographic section: [1] “Do you encounter initiatives aimed at addressing addiction in your daily life?”, [2] “If you were asked to develop a policy to address addiction, what would you do?”, [3] “What are the essential components of an effective substance abuse prevention policy?”, [4] “Do you perceive any shortcomings in current policies?”, and [5] “Is there anything you may have omitted or would like to add?” Necessary precautions were taken to ensure the protection of participants' privacy; no directly identifying information was requested. In reporting the findings, pseudonyms such as Participant 1 (P1, P2, etc.) were used to present quotations and other illustrative examples.

#### 2.2.1 Data quality assurance

Online open-ended surveys carry recognized risks for data integrity, including bot responses, repeat submissions, and mischievous responding. Several safeguards were embedded in the design and conduct of this study to mitigate these risks. The survey was not disseminated through social media or open links; institutional permission was obtained from the university, and the form was distributed to enrolled students at named faculties through the Student Affairs Office, providing an institutionally mediated access pathway. In addition, the research team applied a qualitative form of data screening drawing on cumulative experience with the Turkish university student population, including from prior face-to-face studies. Response sets were examined for substantive coherence across the five open-ended questions, congruence with the lived realities encountered in offline research, and the absence of fabrication markers such as identical wording across participants, off-topic content, or implausibly stylised answers. No such markers were identified. Three participants (P84, P90, P118) submitted entirely blank open-ended responses; rather than removing these silently, we retained them in the analytic record and addressed them reflexively. Brevity and reluctance to engage particularly with the final “anything to add?” prompt are broadly consistent with patterns observed in our face-to-face research on stigmatized topics within this population, and we treat that concordance as supportive rather than corrupting of dataset authenticity. We acknowledge that internal consistency in its strict psychometric sense is a quantitative construct; the procedures described here should be understood as a qualitatively grounded equivalent rather than a psychometric application.

### 2.3 Reflexivity statement

The research team consists of academics specializing in the field of addiction studies. The academic background of the authors of this study tends toward evaluating addiction from a holistic perspective. Consequently, they are closely interested in the public policy dimension of combating addiction, as well as its biopsychosocial facets. As the researchers are affiliated with a university, they conducted the analysis of university students' ideas by maintaining a balance between both an insider and an outsider perspective. For this reason, the authors do not view themselves as passive data collectors of this study. On the contrary, they position themselves as researchers who actively construct meaning. Furthermore, thanks to the researchers' experience in qualitative research, the paradoxical expressions of the students were interpreted beyond being mere contradictions. Throughout the analysis, the team remained reflexively engaged with how their disciplinary backgrounds, prior experience with addiction studies, and academic positioning shaped their reading of the data, treating researcher subjectivity as a resource for interpretation rather than a bias to be eliminated. Particular attention was paid to participants' framings that diverged from the team's initial expectations, and such divergences were treated as analytically generative rather than as anomalies to be reconciled.

### 2.4 Analysis

In the data analysis phase, a descriptive thematic content analysis approach was adopted, consistent with the qualitative descriptive research design as articulated by Sandelowski and elaborated for contemporary health care research practice by Bradshaw et al (16, 22). The qualitative descriptive tradition is particularly well suited to studies aimed at mapping healthcare needs, policy perceptions, and lived everyday concerns at a relatively low level of inferential abstraction. Importantly, this tradition does not entail a purely descriptive or atheoretical stance: qualitative description necessarily involves interpretation throughout, even where the primary aim is to remain close to participants‘ own framings (16). Our analytic approach reflects the principled combination of thematic and content-analytic procedures that Bradshaw et al. (22) identify as appropriate within a qualitative descriptive design. This framing enabled a process of thematisation in which findings were presented directly from the data, with fidelity to participants' own framings, without being filtered through an abstract theoretical lens.

The research team is familiar with the wider thematic analysis literature, including Braun and Clarke's reflexive thematic analysis and their subsequent mapping of TA into reflexive, codebook, and coding reliability variants (23, 24). Because our analytic procedure involves multiple coders, consensus-based theme refinement, frequency reporting, and a descriptively oriented analytic ambition, we judged the qualitative descriptive tradition to be the most coherent home for the present study. As Bradshaw et al. (22) put it, researchers can confidently name their research design as qualitative description, and reference to description does not exclude the exercise of thought, the practice of analysis, the activity of reflection, and the work of interpretation that necessarily occur throughout. Where useful, we have drawn on the iterative cycles of data familiarization, coding, theme development, and theme review that are common across thematic approaches.

The analysis was conducted in stages, with the aim of ensuring methodological transparency and traceability. All responses were first read multiple times by the researchers to develop a general structural understanding of the dataset; notes and observations generated during this reading informed the development of analytic insights. The data were then coded line by line using MAXQDA 2022, yielding a total of 74 codes (25). Coding followed an inductive logic, with sub-themes and themes derived from the data rather than imposed on it (26). Rather than computing a statistical inter-coder reliability coefficient, we relied on researcher reflexivity and peer debriefing approaches that enhance methodological rigor within a qualitative paradigm. After initial coding, the researchers met at regular intervals to discuss the meaningfulness of the codes and the coherence of the emerging thematic structure; the analysis was finalized once consensus was reached.

Findings are presented with supporting frequency values (f), participant identifiers (P1–P119), and direct quotations. The frequency values are not intended to give the findings a quantitative character. They are offered as an aid to reader transparency, allowing the analytic distribution of meanings to be traced across a relatively large written-survey dataset. We recognize that frequencies in qualitative reporting carry interpretative rather than statistical significance, and that the analytic weight of a theme is not reducible to its numerical recurrence.

## 3 Results

Table 1 shows that 72.3% of the study participants were female and 27.7% were male; the average age was 20.66 ± 2.16. 37.0% of the students were in their second year. The students' accommodation is, in order, state-run halls of residence (49.6%), home (43.7%) and private halls of residence (6.7%); they live with their family (38.6%), with a flatmate/roommate (52.9%) or alone (8.4%). The income level of 72.3% of students' families is above the minimum wage, whilst the income level students perceive for themselves is, at the highest rate, 57.1% at a middle level. Furthermore, the educational background of the families is as follows: the majority of mothers (32.8%) are primary school graduates, whilst the majority of fathers (37.8%) hold a bachelor's degree or higher.

**Table 1:** Findings on the sociodemographic characteristics of students.

Variables	Variables	*%* (n)
**Sex**	Male	27,7 (33)
	Woman	72,3 (86)
**Class level**	Preparation	0.8 (1)
	1st year	33,6 (40)
	2nd year	37,0 (44)
	3th year	21,0 (25)
	4th year	6,7 (8)
	5th year	0.8 (1)
**Place of residence**	Public dormitory	49,6 (59)
	Private dormitory	6,7 (8)
	Home	43,7 (52)
**People they live with**	Roommate / Housemate	52,9 (63)
	With family	38,6 (46)
	Alone	8,4 (10)
**Number of siblings**	None	8,4 (10)
	1	33,6 (40)
	2	31,1 (37)
	3	16,0 (19)
	4 or more	10.9 (13)
**Family income level**	Below minimum wage	7,6 (9)
	Minimum wage	20,2 (24)
	Above minimum wage	72,3 (86)
**Perceived income level**	Low	36,1 (43)
	Medium	57,1 (68)
	High	6,7 (8)
**Place of birth**	Village	9,2 (11)
	District / Town	25,2 (30)
	City / Province	65,5 (78)
**Mother's educational status**	Not literate	2,5 (3)
	Literate (no formal schooling)	0,8 (1)
	Primary school	32,8 (39)
	Middle school	12,6 (15)
	High school	26,9 (32)
	Bachelor's degree or higher	24,4 (29)
**Father's educational status**	Not literate	0,8 (1)
	Literate (no formal schooling)	1,7 (2)
	Primary school	21,0 (25)
	Middle school	13,4 (16)
	High school	25,2 (30)
	Bachelor's degree or higher	37,8 (45)
**Mother's occupation**	Unemployed	65,5 (78)
	Self-employed	5,0 (6)
	Worker	9,2 (11)
	Civil servant / Public employee	10,9 (13)
	Retired	9,2 (11)
**Father's occupation**	Unemployed	5,9 (7)
	Self-employed	10,1 (12)
	Worker	27,7 (33)
	Retired	39,5 (47)
	Civil servant / Public employee	14,3 (17)
	Deceased	2,5 (3)

*n*: Count.

### 3.1 Experience of encountering addiction prevention policies

The question posed to participants, “Do you encounter measures aimed at addressing addiction in your daily life?”, yielded meaningful findings from both quantitative and qualitative perspectives. More than half of the participants (55.5%, *n* = 66) reported that they had not encountered such measures (Table 2). Content analysis of the responses from the 44.5% of participants (*n* = 53) who reported encountering such measures revealed a notable pattern (Table 3). The presence of a friend or family member with an addiction, personal experiences, or accounts of addiction within their immediate social circle constituted a prominent domain of exposure to this issue (*n* = 17). Activities in educational settings (*n* = 12), posters and banners (*n* = 9), seminars/conferences (*n* = 9), and public service announcements (*n* = 8) may be considered encounters facilitated through institutional efforts. In addition, social media content also represents a domain of exposure (*n* = 7). However, the number of direct references to key institutions and organizations involved in addressing addiction was limited to six participants.

**Table 2:** Exposure to addiction prevention practices.

Category	*n*	%
Not Exposed	66	55.5
Exposed	53	44.5
Total	119	100

**Table 3:** Areas identified by participants with exposure experience.

Area of exposure	*n* ^*^
Personal experience (friends/family/social circle)	17
Educational setting	12
Posters	9
Seminars	9
Public service announcements	8
Social media	5
Package warnings	4
Healthcare institutions	3

^*^As some participants mentioned more than one area, the total exceeds n = 53.

Some participants expressed certain criticisms even within their brief responses to this question. For example, one participant (P34) stated that awareness campaigns “failed to attract attention,” while another (P81) remarked that cigarette package warnings “did not work very well.” These findings suggest that the social visibility (or memorability) of anti-addiction initiatives remains limited, and that current communication strategies may be insufficient in reaching broader audiences. In this context, the policy recommendations derived from the qualitative analysis presented later in the findings may be considered particularly meaningful. This is because, in the presence of policies that lack sufficient visibility or memorability, the perspectives of young students may offer relatively unbiased interpretative and suggestive contributions.

### 3.2 Policy recommendations and expectations: findings of the thematic analysis

A comprehensive analysis of the responses to the second, third, fourth, and fifth questions identified eight main themes. Table 4 presents the themes, sub-themes, frequency values of the coded content for each sub-theme, and illustrative quotations.

**Table 4:** Thematic analysis: themes, sub–themes, and sample quotations.

Theme	Sub–theme	f	Sample quotation
Prohibitions, sanctions, and monitoring mechanisms (*f* = 76)	Reducing access	16	“*I would try to minimize accessibility as much as possible*.” P13
	Increasing inspections	14	“*I believe there would be improvement if inspections were conducted more effectively*.” P9
	Strict prohibitions	13	“*I would ensure public order in every area where sales take place*.” P108
	Supply–oriented interventions	13	“*I would eliminate the issues that drive people toward addiction*.” P5
	Increasing sanctions	12	“*I would increase penalties and ban entry into the country as well as production*.” P3
	Controlled purchasing policy	5	“*I would implement a controlled purchasing policy*.” P16
	Smoke–free zones	3	“*Smoking bans near institutions fall under this scope*.” P9
Education and awareness–oriented approaches (*f* = 74)	Increasing education and awareness	37	“*I would raise individual awareness through education and outreach activities*.” P7
	Family–based and early childhood interventions	16	“*I would prioritize programs and awareness efforts specifically targeting families*.” P19
	Deterrence: emphasizing harms	8	“*The harmful aspects of the addictive substance should be highlighted*.” P6
	Positive anti–addiction discourse	6	“*Showing how many beautiful things there are in life*.” P21
	Preventing early onset	6	“*I would like to conduct studies to eliminate addiction among minors*.” P30
Policy characteristics (*f* = 71)	Inclusivity	13	“*Targeting only punitive measures and consequences is insufficient*.” P23
	Sustainability	12	“*I would establish sustainable, phased systems*.” P12
	Feasibility	9	“...I would try to develop a more realistic and feasible policy that is not simplistic.” P51
	Accessible support	9	“*I would have directed people who cannot access support*.” P16
	Preventative and curative	6	“*I would adopt a preventative and curative approach*.” P7
	Determination and willpower	5	“*Building and strengthening an individual's willpower is very important*.” P36
	Addressing new types of addiction	4	“*Policies regarding behavioral addictions such as technology, shopping, and gambling are insufficient*.” P31
	Regular follow–up and support	3	“*There are shortcomings regarding long–term observation*.” P72
	Trustworthiness	3	“*If the addicted person does not trust the policy, the logic of the policy fails*.” P64
	Holistic approach	3	“*I would follow a curative and holistic path*.” P7
Treatment and psychosocial support services (*f* = 60)	Psychological support	16	“*I would make psychological support mandatory*.” P16
	Early intervention through screening programs	9	“*I would ensure they receive support by establishing early screening and intervention systems*.” P7
	Social support	9	“*Creating a space where individuals with addiction can come together and interact*.” P30
	Post–treatment social support	8	“*Sufficient support is not provided regarding reintegration into society after treatment*.” P31
	Healthcare–based interventions	7	“*I would like healthcare practices to be more clearly regulated*.” P27
	Establishing combatting/treatment centers	5	“*A center related to combatting addiction could be established*.” P9
	Competent psychosocial support	4	“*Qualified experts in the field, necessary financing, and steady progress*.” P57
Humanistic and empathetic approaches (*f* = 52)	Supportive attitude	8	“*Supporting them under any circumstances and not judging them*.” P68
	Environment for safe disclosure	7	“*Maybe the solution is not to block the paths they escape to*.” P34
	Empathetic attitude	7	“*Accepting the addicted person humanely and making them feel valued*.” P83
	Restorative and curative approach	7	“*I would follow a restorative, curative, and holistic path, not just a punitive one*.” P7
	Person–centered policies	6	“*Using a method directed toward the individual before you*.” P32
	Considering lived experiences of addiction	4	“*Ensuring [policies] are carried out by people who have truly struggled with addiction*.” P47
	Developing love and understanding	3	“*At its core, there are deficiencies originating from the family; love and understanding come first*.” P54
	Respect	3	“*Addicts are viewed as monsters*.” P96
	Other (Human rights, volunteerism, etc.)	7	“*Accepting the addicted person humanely*.” P83
Criticisms of current policies and lack of knowledge (*f* = 38)	Unfamiliarity with existing policies	17	“*The lack of awareness regarding policies might also be a deficiency*.” P13
	Lack of feasibility	6	“*Even if there are good policies in theory, there are many shortcomings in practice*.” P6
	Perceived inadequacy of current policy	4	“*I think it is generally insufficient*.” P11
	Lack of preventative focus	3	“*Preventative efforts are not strong enough*.” P7
	Judgmental nature [of policies]	3	“*I would focus more on understanding young people rather than judging them*.” P71
	Ease of access (criticism)	3	“*The fact that access to drugs is very easy increases addiction*.” P9
Social participation and alternative activities (*f* = 35)	Increasing social participation	11	“*If you close down social areas, addiction increases*.” P2
	Social interaction	7	“*I would encourage the active participation of families and local governments in the process*.” P7
	Economic development	7	“*Increasing people's welfare levels could be a policy*.” P11
	Fostering positive habits	3	“*We should be able to replace harmful substances with things that provide benefit*.” P9
	Other (Habit change, etc.)	7	“*I would establish small places in every neighborhood for people to spend time together*.” P38
Research and youth participation (*f* = 12)	Increasing addiction research	9	“*Identifying the root causes of the problem, working on its causes and mechanisms, and being informed by individuals from all segments of society*.” P58
	Increasing student recommendations	2	“*I suggest preparing policy documents with the participation of students*.” P37
	Policies targeting specific groups	1	“*It is essential that the target group is included when developing policy*.” P37

### 3.3 Prohibitions, sanctions, and monitoring mechanisms

This theme encompasses participants' proposals regarding the restriction of access to addictive substances, the intensification of monitoring efforts, and the strengthening of criminal sanctions. The core components of this theme consist of reducing accessibility, enhancing oversight, implementing strict prohibitions, and executing supply-oriented interventions.

A significant proportion of participants identify the reduction of accessibility as a primary strategy. This perspective is encapsulated in P13′s statement: “I would try to minimize accessibility as much as possible.” Some participants, such as P108, adopted a more resolute stance, asserting, “I would ensure public order in every area where sales take place; I believe this is the definitive solution.” Meanwhile, P3 proposed a dual approach - combining punitive measures with supply-side restrictions–stating, “I would increase penalties and ban both the import and production [of these substances].”

However, alternative perspectives were also voiced. For instance, P26 highlighted the supply-demand balance, stating, “In my view, the intervention should address the root causes and ensure minimal product availability, rather than simply making access difficult.” This viewpoint illustrates that while some individuals believe prohibitionist approaches alone may be insufficient, they nonetheless emphasize the critical importance of prevention.

### 3.4 Education and awareness-oriented approaches

This theme encompasses recommendations regarding public awareness campaigns, school-based educational programs, and family-focused awareness initiatives. The primary sub-thematic components of this theme consist of education and awareness-raising, family-centered interventions, deterrence through the highlighting of harms, and early-age prevention.

The vast majority of participants perceive education as the primary policy instrument. P7 stated, “The awareness of individuals and society should lie at the heart of a robust anti-addiction policy,” while P24 remarked, “I believe education is consistently the most vital factor; individuals should be informed about the extent of the harm that addictive substances can cause.”

In a notable observation, P55 emphasized the responsibility of educators and those shaping addiction discourse to serve as role models: “It is contradictory that the individuals conducting this work are generally school teachers or healthcare professionals; while it is widely known that substance abuse is harmful, these individuals themselves often exhibit high levels of tobacco and alcohol consumption.” This statement serves as a significant critique regarding the perceived inconsistency in the conduct of those implementing policy.

### 3.5 Policy characteristics

This theme reflects perspectives on the essential attributes that an effective substance abuse prevention policy should possess. Its core components comprise inclusivity, sustainability, feasibility, accessibility, and a holistic approach.

Consistent with the general framework of this theme, P52 emphasized the importance of policy sustainability, stating: “It must be continuous, and monitoring must be carried out with genuine care; furthermore, analyses and solutions must be realistic, based on the results achieved.” Meanwhile, P64′s statement underscores the concept of trust: “I believe trust is paramount. If the individual struggling with addiction does not trust the provider or the policy itself, the policy loses its fundamental logic.”

A notable finding was the identified deficiency in policies addressing emerging forms of addiction, which was highlighted as a significant concern. For instance, P31 emphasized the necessity of expanding beyond the traditional focus on substance use disorders, stating: “The fight against addiction is currently confined to substance-related issues; however, behavioral addictions-such as technology, shopping, and gambling-are increasingly on the rise.”

### 3.6 Treatment and psychosocial support services

This theme emerged from discussions encompassing recommendations for psychological support services, early intervention programs, social support mechanisms, and post-treatment reintegration processes.

P20 emphasized the accessibility of psychological support, proposing the establishment of “easily accessible psychological support and rehabilitation centers for individuals struggling with addiction.” While deficiencies in the post-treatment phase were identified as a significant area of concern, participants expressed a clear need for enhanced support in this regard. For instance, P31 drew attention to these expectations by criticizing the “lack of sufficient support for reintegrating individuals into society following the treatment process.”

Competence within the framework of combating addiction was also reflected in the statements of several participants. P57 underscored the necessity of merit and professional expertise, stating: “Qualified experts in the field, necessary funding, and steady progress,” thereby highlighting the critical importance of the quality of policy implementers.

### 3.7 Humanistic and empathetic approaches

This theme encompasses perspectives on the significance of maintaining a non-judgmental, supportive, and empathetic attitude toward individuals struggling with addiction. The core components of this theme consist of a supportive stance, an empathetic approach, restorative policies, and a person-centered understanding.

During the analysis, it was particularly striking to observe that individuals championing this view often had personal experience with addiction, either personally or through close relatives. This finding indicates that individuals with lived experience tend to employ a more empathetic and restorative discourse in their policy recommendations, highlighting the critical nature of this expectation.

For instance, P68 emphasized the importance of a non-judgmental attitude, stating, “To support them under all circumstances and not to judge them.” The discourse regarding the inclusion of lived experiences -particularly within the policymaking process- is noteworthy. In this context, P47 proposed involving those who have firsthand experience with addiction in policy development: “It should be carried out by people who have genuinely struggled with addiction.” Furthermore, P54′s statement reflects a profound humanistic insight: “I observe that at the root of most people's addiction lies a deficiency stemming from the family. Love and understanding come first.”

### 3.8 Criticisms of current policies and lack of awareness

This theme encompasses both criticisms directed at existing policies and the participants‘ self-reported lack of knowledge regarding them. The sub-components of this theme consist of a lack of awareness of policies, implementation challenges, the failure of policies to be sufficiently preventive, and a perceived judgmental attitude. In this context, the participants' occasional self-critical reflections appear to be linked to the awareness-raising effect of such research studies.

For instance, P13′s self-critical observation is particularly noteworthy: “I cannot comment because I lack knowledge about current policies. However, noting that a lack of knowledge regarding these policies might be a deficiency does not generate enough of an impact.” This statement suggests that it is not merely the existence of policies but their social visibility that constitutes a critical issue.

Meanwhile, P6 highlighted the divide between theory and practice: “Even if there are sound policies in theory, there are numerous shortcomings in practice.” The judgmental tone attributed to policies also emerged as a significant point of criticism. P71 offered both a critique and a recommendation simultaneously, stating: “I would focus more on understanding young people rather than judging them.” Furthermore, P96 drew attention to the extent of social stigmatization with the remark: “Addicts are viewed as monsters.”

### 3.9 Social participation and alternative activities

This theme centers on proposals aimed at increasing social participation, establishing spaces for social interaction, improving economic conditions, and fostering positive habits.

P2 highlighted the social dimension of addiction, stating: “Addiction is a process. If you close down social spaces, addiction to cigarettes, alcohol, and drugs increases and becomes normalized.” Meanwhile, P38 offered a concrete suggestion: “I would establish small spaces in every neighborhood where people could spend time together.”

The role of economic factors emerged as a frequently emphasized theme. P58 remarked, “The real issue relates to the decline in social welfare and the fact that society is becoming increasingly impoverished in class terms due to economic crises,” while P11 highlighted the role of economic development in combating addiction by stating, “Improving people's standards of living could be a policy.”

### 3.10 Research and youth participation

This theme emerged from recommendations concerning the expansion and support of addiction research, alongside the active involvement of young people in policymaking processes.

P37 put forward an original proposal highlighting the significance of student participation in the policymaking process: “I propose drafting policy documents with the direct participation of students. With clear guidelines, a comprehensive study could be produced. I would also provide researchers with funding and resources to identify current addictions.” This proposal offers valuable insights from the perspectives of both participatory policy development and an evidence-based approach.

## 4 Discussion

This study was conducted to ascertain and evaluate the perspectives and recommendations of university students regarding substance abuse prevention and its associated policies. In addition to the sociodemographic characteristics of the participants, their policy recommendations and expectations concerning substance abuse prevention were analyzed under eight distinct themes and 52 sub-themes. To the best of our knowledge, this study represents the first qualitative inquiry to present university students' views on substance abuse prevention strategies within a comprehensive thematic framework, distinguishing itself from existing literature through its substantial sample size. Furthermore, the study offers model proposals for both punitive and rehabilitative measures from a student-centered perspective. Consequently, it is posited that this research contributes to the literature by providing empirical data on potential innovations and improvements to substance abuse policies from a youth-oriented viewpoint. Additionally, alongside the call for youth participation in decision-making processes, the study provides valuable insights for both the academic community and policymakers.

In our study, 55.5% (*n*=66) of participants reported that they had not encountered any initiatives aimed at combating addiction in their daily lives. The high number of individuals reporting a lack of exposure to any anti-addiction applications or programs at any stage of their lives suggests that the prevalence and accessibility of such efforts remain limited. Furthermore, this situation indicates an insufficiency in awareness and knowledge regarding the issue among the university students included in our research. This finding aligns with previous studies reporting that awareness levels regarding addiction among university students remain constrained (27, 28). Among those who had encountered anti-addiction efforts at some point in their lives, the fact that personal life experiences (family, friends, close social circles, etc.) were the most frequent source of exposure suggests that young people's knowledge and awareness of addiction are primarily shaped by their micro-level social environments. Bandura's Social Learning Theory, which plays a significant role in the development of addiction and is addressed by numerous studies, also manifests in the processes of addiction prevention and combat. According to this theory, social learning mechanisms are prominent (29). The findings obtained from our study emphasize the importance of social learning in the context of combating addiction. Additionally, while this finding suggests that proactive and structured prevention activities may be inadequate, the fact that educational settings, posters, and public service announcements follow personal experience as sources of information may indicate that a vast majority of young people perceive these activities reactively, as well as demonstrating that existing applications are indeed capable of reaching youth.

The high frequency of the theme ”Prohibitions, Sanctions, and Monitoring Mechanisms“ in our research suggests that participants prioritize macro-level intervention systems in the fight against addiction; consequently, it can be stated that students advocate for a restriction- and authority-oriented approach. Furthermore, the fact that the ”Reducing Access“ sub-theme holds the highest frequency under this theme can be interpreted as young people viewing the obstruction of access to addictive substances as one of the most effective ways to prevent addiction. The statement by P13, “I would try to minimize accessibility as much as possible,” corresponds to the discourse of supply reduction from the students' perspective. Corroborating these views, various studies emphasize that strategies limiting access and preventing contact with substances are among the most crucial practices in reducing and preventing the development of addiction (30, 31). Similar to the emphasis on supply reduction in P13′s statement, the remark by P3, “I would increase penalties and ban its entry into the country as well as its production,” can also be considered a discourse aimed at supply reduction. Therefore, by reporting that penalties should be increased, participants emphasized that addiction could be prevented through supply reduction, just as with the reduction of access. Moreover, the demands for “Strict Prohibitions” expressed by the students in our study- similar to the “Increasing Sanctions” sub-theme-highlight the deterrent effect of penalties in addiction prevention. The statement by participant P108, “I would ensure public order in every area where sales take place,” suggests that the students in our study perceive addiction as a security issue that requires stringent control. Studies examining youth perspectives on anti-drug policies show that increasing penalty rates and tightening monitoring mechanisms receive significant support among young people (32, 33). In a study conducted in Türkiye, while young people identified “policies increasing penalty rates” as the most effective option after supply-reduction policies, Australian youth also frequently proposed harsher sanctions and a more intensive police presence against alcohol and other drug problems (34, 35). Conversely, some studies in the literature have noted that young adults find the use of oppressive and punitive policies ineffective in combating addiction (36, 37). This discrepancy may primarily stem from socio-cultural variations and policy-related differences between countries and individuals.

Another prominent emphasis among our study findings belongs to the ”Increasing Inspections“ sub-theme. The statement by participant P9, “I believe there would be improvement if inspections were conducted more effectively,” highlights a potential deficiency in current practices. Consequently, it can be stated that the students in our study view the monitoring and follow-up of existing policies as a critical step in combating addiction, rather than merely focusing on the development of new policies. This situation can be explained by the gap between law and implementation addressed in various studies. Similar to the participants in our research, existing literature suggests that the discrepancy between legislation and enforceability can be mitigated through rigorous inspection (38). In addition to their macro-level perspectives, the students' thoughts expressed through sub-themes such as “Smoke-Free Zones” and “Controlled Purchasing Policy” demonstrate their belief that environmental regulations play a critical role in supporting individual behavioral change.

It can be stated that the students in our study perceive the theme of ”Education and Awareness-Oriented Approaches“ as being as vital as policies and legal regulations in the development of addiction-fighting strategies. In another study conducted among university students, it was found that 48.6% of students had not previously received any addiction-related education, and these students exhibited low levels of awareness regarding substance abuse (39). Another research indicated that students participating in an educational program on combating addiction showed an increase in both their knowledge levels regarding addictive substances and their refusal skills; furthermore, it was determined that misconceptions and false beliefs concerning addictive substances decreased (40). Similarly, it has been reported that trainings provided in the field of combating addiction lead to significant improvements in the awareness and attitude levels of university students (41). Additionally, positive changes in university students' beliefs regarding substance use were noted following a Health Belief Model-based education, even when not specifically focused on addiction (42). There is an extensive body of literature demonstrating that education-based interventions targeting youth in the field of addiction possess protective and preventive effects. In this context, it can be said that our findings align with the literature, and the fact that young people prioritize education and awareness efforts indicates a strong perception regarding the effectiveness of such interventions. In light of these results, it is recommended to ensure the continuity of education and awareness-based activities in the fight against addiction and to expand these efforts through institutional support.

It is evident in the sub-themes of “Family-Based and Early Childhood Interventions” and “Preventing Early Onset” that the participants in our study tend to advocate for shifting addiction prevention efforts to the early stages of life. Here, the response provided by participant P19, “I would prioritize programs and awareness efforts specifically targeting families,” along with the statement by P30, “I would like to conduct studies to eliminate addiction among minors,” support the interpretation that participants hold an ecosystemic approach. Correspondingly, the literature emphasizes that strengthening parent-child communication and raising awareness among families, particularly within the context of universal prevention, serve as one of the most critical keys to protecting individuals from addiction (43, 44).

Another significant finding of our study pertains to the comments categorized under the theme of “Policy Characteristics,” through which participants evaluated the quality of addiction prevention policies. The high frequency of comments in this section suggests that young people support interventions focused on authoritarian and structural restrictions in the fight against addiction. The results of our study emphasize the necessity of inclusive and sustainable structural policies for an effective response to addiction. The remarks by participant P23, “Targeting only punitive measures and consequences is insufficient,” and P12, “I would establish sustainable, phased systems,” underscore the need to develop policies that encompass every stage of the addiction-combating process and incorporate long-term, systematic structures rather than short-term solutions. Furthermore, it was shared that policies should not merely occupy a judicial position but should also take on a preventive and curative role. In this regard, the social dimension of addiction-fighting policies was emphasized. Alongside the sub-themes of “Feasibility” and “Accessible Support,” these views highlight the prioritization of integrating individuals struggling with addiction into the system and implementing practices based on realistic approaches. Students also emphasized the emerging needs regarding new types of addiction. Coupled with this perspective, the importance of considering the dynamics and needs of contemporary society was shared. Research highlights that success in combating addiction is enhanced by the integration of restrictive measures and screening and brief interventions by healthcare professionals alongside education (45–47). Additionally, studies in the literature support the view that policies which are socio-culturally sensitive and feasible provide greater societal benefit (48). Moreover, it is stated that removing barriers to access is vital for addiction prevention and treatment policies to reach all segments of society, and scalable interventions should be prioritized to this end (49).

The significance of the theme ”Treatment and Psychosocial Support Services“ can be inferred from its high frequency of representation among the university students in our study. Within this theme, the ”Psychological Support“ sub-theme - which carries a markedly higher frequency compared to others - suggests that students believe it is essential to approach addiction as a process requiring mental health support. This perspective is clearly evidenced in the statement by participant P16, who remarked, ”I would make psychological support mandatory.“ To prevent addiction before it begins, students in our study proposed interventions such as “Early Intervention Through Screening Programs” and “Establishing Combatting/Treatment Centers.” Research emphasizes that screening, brief Intervention, and referral to treatment practices are invaluable for identifying at-risk individuals, increasing treatment engagement through brief interventions, and preventing relapse (50, 51). Consequently, as with other medical conditions, the necessity of early detection and intervention in addiction can be viewed as a vital component of the struggle. A meta-analysis reported that early interventions against substance use can result in reduced substance consumption and improved behavioral outcomes (52). In our study, it was similarly found that students recommended prioritizing this issue within a framework of effective treatment and psychosocial support services. Furthermore, the sub-themes of “Social Support” and “Post-treatment Social Support” reveal a prevailing view among students that treatment extends beyond the clinical dimension and must be approached holistically. Studies in the literature have proven that social support networks both shape risk and protective factors regarding substance use and enhance treatment efficacy while preventing relapse (53). One study found an inverse correlation between university students' perception of the future and hope levels and their tendency toward substance use; specifically, students with hopeful and positive expectations for the future exhibited a lower tendency toward substance use (54). Another study demonstrated that the risk of substance use increases in individuals with low levels of future-oriented thinking and planning (55). In parallel with these findings, the results obtained from our study suggest the importance of psychosocial support, socialization, rehabilitation efforts, hope, and belief in the future, as well as the necessity for policies that incorporate or support these elements. Finally, through the sub-themes of “Competent Psychosocial Support” and “Healthcare-based Interventions,” students offered suggestions and views regarding the quality of addiction services, expressing the belief that these services can only achieve success when administered by experts in the field with clearly defined boundaries.

The findings of our study re-emphasize that the fight against addiction encompasses two distinct axes. While one of these domains involves sanctions aimed at supply reduction, the other consists of individual-centered “Humanistic and Empathetic Approaches” on the demand side. Considering the total frequency weights of both the “Prohibitions, Sanctions, and Monitoring Mechanisms” and “Humanistic and Empathetic Approaches” primary themes, it can be argued that the university students included in our study seek effective solutions for addiction through both internal and external processes. The fact that participants generally expect a rigorous intervention from the state alongside a humanistic approach can be explained through the Socio-Ecological Model. Since each subsystem operates in coordination with one another, this interconnectedness is reflected in the views and attitudes of individuals. Through the sub-themes of “Supportive attitude” and “Empathetic Attitude,” it can be stated that students perceive addiction as a health and adaptation issue. P96′s statement, “Addicts are viewed as monsters,” aligns with the prevalent social stigmatization of individuals with addiction frequently cited in the literature (56). Furthermore, our results reveal that university students believe a restorative and curative method should be integrated into addiction-fighting strategies. This sentiment is clearly reflected in P7′s statement: “A restorative, curative, and holistic path, not just a punitive one.” Consequently, while the students in our study expect this process to be managed at the macro level through punitive systems, they simultaneously advocate for it to be supported by environments where humanistic values- such as empathy, support, respect, and restoration - are prioritized. Research frequently emphasizes that compassion-based approaches constitute the foundational step for improvement at both individual and structural levels (57).

The lack of sufficient recognition regarding existing practices and policies constitutes one of the most significant structural issues and barriers in the fight against addiction. This situation is highlighted through the theme of “Criticisms of Current Policies and Lack of Knowledge” in our study. The sub-theme of “Unfamiliarity with Existing Policies,” as reported by the university students in our research, suggests that the initiatives implemented by legislators may not have sufficiently reached the target population. Furthermore, this finding supports the view that there is a deficiency in knowledge concerning addiction-related policies among the students included in our study. Participant P13 demonstrated agreement with this perspective, stating: “The lack of awareness regarding policies might also be a deficiency.” Research indicates that increasing youth awareness of policy issues through educational settings can reduce skepticism toward the system and garner trust (58, 59). Additionally, the criticism of a “Lack of Feasibility,” when combined with the emphasis on monitoring found in previous themes, serves to demonstrate that participants do not desire policies that exist merely on paper; rather, they prioritize strategies that are effectively integrable into the field.

It can be argued that participants perceive addiction as a leisure and welfare-related issue, as evidenced by their responses under the theme of “Social Participation and Alternative Activities.” Through the statements of participant P2, “If you close down social spaces, addiction increases,” and participant P9, “We should be able to replace harmful substances with things that provide benefit,” it is suggested that there is an emphasis on the necessity of providing alternative arrangements that can replace addictive elements for individuals. Consequently, this demonstrates that providing such alternatives through positive habits and economic development opportunities can serve as a permanent solution for the prevention of addiction and relapse. Research indicates that this practice, referred to as substitution-based intervention, contributes to improved outcomes (60).

The findings of our study reveal the importance of including the target group in the preparation process of addiction-fighting strategies while those strategies are being developed. Furthermore, it highlights the necessity of integrating scientific data into this process. Consequently, the theme of “Research and Youth Participation” in our study emphasizes the significance of conducting addiction-combating efforts through a participatory model. Through the statement of participant P58, “Identifying the root causes of the problem, working on its causes and mechanisms, and being informed by individuals from all segments of society” it is suggested that there is an emphasis on the importance of developing evidence-based solutions. Thus, it can be stated that some students in our study advocate for the importance of clarifying the complex nature of addiction. Additionally, some students in the study expressed a desire to be involved in policy-planning processes. Research indicates that involving young people in program and policy design not only contributes to the adoption of the process but also supports the enhancement of trust in the system (61).

This study has several limitations. First, the data collection method based on written surveys may remain limited in terms of depth of content compared to face-to-face interviews. A reluctance to provide detailed content was observed in some participants (e.g., P12), and three participants (P84, P90, P118) provided entirely blank responses. Second, the study group is limited to students from a single university. Furthermore, in accordance with the general characteristics of qualitative research, our findings do not claim generalizability. Third, the online data collection method restricted the researcher's ability to ask probing questions. However, the relatively large number of participants provided an advantage in terms of thematic diversity; the findings offer a roadmap regarding which areas and with which individuals in-depth interviews should be conducted in future studies. Additionally, the statements of some participants (e.g., P47) suggesting that the experiences of individuals specifically struggling with addiction should be revisited have encouraged us to pursue face-to-face, in-depth studies in this context.

### 4.1 Policy recommendations and implementation

- In light of the data obtained from the participants, the following strategies are proposed to make addiction policies more effective and youth-oriented:
- It is considered that maintaining the fight against addiction through a centralized structure, rather than a fragmented one, would be more effective. Therefore, the establishment of an addiction coordination center with an interdisciplinary organizational structure is recommended. This would ensure the sustainability of policies while simultaneously preventing data loss and deficiencies.
- When developing policies regarding the fight against addiction, it is recommended to adopt a “substance-agnostic” approach-one that encompasses all types of addiction. Furthermore, care should be taken to ensure that these policies are inclusive, applicable, sustainable, and accessible. Accordingly, they should be designed to be accessible in a way that reaches disadvantaged groups in peripheral regions.
- It is recommended to identify and disseminate standardized screening programs that support the early detection of risky situations at every stage of education, starting from primary school, as well as within local neighborhoods.
- Young people should not only be the target audience of addiction policies but should also be seen among their architects. Therefore, it is recommended to prioritize the views of young people in policy-making processes. Additionally, supporting scientific and volunteer activities in the fight against addiction-particularly those involving youth-and creating resources in this field should be adopted.
- Social, cultural, and athletic alternative living spaces that can serve as substitutes for addiction should be created for everyone, especially for children and youth at risk; these activities should not be limited to urban centers but should be extended to peripheral regions.
- A common terminology that is supportive, restorative, and does not marginalize individuals with addiction should be developed for both the public sphere and service delivery. It is recommended to adopt a discourse that emphasizes the harmful aspects of addiction while highlighting the positive facets of life and health, and simultaneously supporting the self-efficacy of individuals during educational activities.
- Large-scale information campaigns should be supported to introduce and deliver existing practices to all segments of society.
- Young people specifically emphasize the importance of a safe environment. Therefore, in accordance with these demands, prohibitions regarding substance supply should be tightened, and criminal sanctions and inspections should be increased.
- It is recommended to increase the number of institutions providing mental and social support that are accessible to all segments of society both before and after the onset of addiction.
- It is recommended to strengthen domestic protective factors through informative and awareness-raising activities aimed at families.

## 5 Conclusion

The thematic analysis findings reveal that the university students in our study possess a multidimensional perspective regarding addiction prevention policies and practices. The fact that “Prohibitions and Monitoring Mechanisms” and “Education and Awareness-Oriented Approaches” reached the highest frequencies demonstrates that participants consider both restrictive and preventive strategies to be essential.

The finding regarding the lack of awareness of existing policies indicates that social visibility, rather than merely the existence of policies, is a critical success criterion. Furthermore, the findings under the theme of “Humanistic and Empathetic Approaches” emphasize the importance of involving individuals with lived experience of addiction in the policy development process.

These findings offer a roadmap for future research concerning the specific areas and individuals with whom in-depth interviews should be conducted. In particular, in-depth interviews conducted with participants who have lived experience of addiction, alongside youth perspectives, could provide invaluable contributions to policymaking processes.

## Data Availability

The raw data supporting the conclusions of this article will be made available by the authors, without undue reservation.
